# The Use of Insect Pigment in Art Works

**DOI:** 10.3390/insects15070519

**Published:** 2024-07-10

**Authors:** Ayça Alper Akçay

**Affiliations:** Faculty of Fine Arts, Atatürk University, Erzurum 25240, Türkiye; ayca.alper@atauni.edu.tr

**Keywords:** art, carmine, Cochineal insect, *Dactylopius coccus*, red pigment

## Abstract

**Simple Summary:**

From Ancient Egypt to the present day, acquiring the color red in the art world has involved various methods. Over the centuries, these methods have included oxides, natural minerals, and organic dyes obtained from plants and insects. This study focuses on the Cochineal insect, which produces a natural red pigment called carmine. This pigment has been used in art for thousands of years, especially by indigenous cultures for textile dyes and paintings. During the Renaissance, artists valued carmine for its bright red tones. The study examines the history of the Cochineal insect, its role in art, and its modern perception. It also discusses famous artworks created with cochineal dyes and how these pigments have inspired artists. Paints obtained from insects are generally regarded as a natural and organic source, making them an environmentally friendly option. Incorporating insect-derived paints into artworks allows for the integration of an organic element sourced from nature. Furthermore, the use of these paints can help enrich artworks aesthetically and conceptually. In conclusion, the use of insect-derived paints depends on the artist’s preferences and the messages they aim to convey through their works.

**Abstract:**

In this compilation, the focus is on the Cochineal insect (*Dactylopius coccus* Costa, 1835 (Hemiptera: Dactylopiidae)), a creature native to South America that produces a potent natural red pigment known as “carmine”. This pigment, utilized for obtaining the color red, has been an integral part of the art world for thousands of years. Indigenous cultures, in particular, have employed the dye extracted from this insect in the creation of textile dyes and paintings. Moreover, the Cochineal insect and its unique pigments have not only supported artistic expression but also captivated and inspired artists. During the Renaissance period, artists preferred the carmine pigment produced by the females of the Cochineal insect for obtaining bright and vivid red tones. This study delves into the history of the Cochineal insect, its role in art, and its perception in the modern world. Famous paintings created with dyes obtained from the Cochineal insect are discussed, exploring how pigments have found a place in the art world and how artists have utilized this extraordinary source to create distinctive works.

## 1. Introduction

Art is a manifestation of humanity’s emotional expression and creativity, and throughout history, artists have expressed their emotions, narrated the human experience, and interpreted the world using the power of colors and symbols. These colors and symbols not only serve as forms of artistic expression but also hold historical, cultural, and scientific significance. Examining these special tools in the color palette of the art world not only enriches our understanding of art but also provides insights into the history of humanity.

Our world is filled with natural elements, such as the sky, earth, water, and fire, each of which is rich in different colors. Throughout history, humans have noticed this diversity and made efforts to see and recreate these colors in their surroundings. This is a natural instinct because color is a product of light, which is the source of life. However, the challenge of finding suitable materials to produce permanent colors in our environment has captivated human curiosity and effort from prehistoric times to the present day. Color is a quality perceived by the eyes as a result of the reflective or emissive properties of an object’s surface [1]. Color arises from how objects or surfaces reflect or absorb light and is experienced by humans through visual perception. The source of colors is usually the result of the reflection or absorption of light. Colors are formed by light appearing at different frequencies at various wavelengths of the electromagnetic spectrum. Light produces different colors in different regions of the electromagnetic spectrum [2]. The three primary colors forming the basis of these colors are red, green, and blue, and they are referred to as primary colors [3]. Colors are created by pigments or substances present on the surfaces of objects. These pigments allow objects to appear in a certain color by reflecting or absorbing light at specific wavelengths. For example, an object appearing red is the result of the pigments on its surface reflecting red light and absorbing light in other wavelengths. The source of colors is related to the characteristics of light and the interaction of pigments on the surfaces of objects. Enriching color sources with materials found in nature, such as rocks, plants, and animals, has been a focal point of interest in art and design studies from the past to the present.

Animals, including both vertebrates and invertebrates, can exhibit extraordinarily colorful appearances. There are several aspects to this color diversity. Color is produced either structurally or by pigments in the environment [4]. Structural coloration constitutes a significant part of the phenotype of many animals [5]. Definitions of the physical mechanisms of structural color production are diverse and often repetitive [6], but structural colors typically arise from the refraction, scattering, or, more commonly, interference of light by layers of modified setae or scales, or surface shaping [7]. For example, the white prosoma of the *Argiope* genus (Araneidae) appears to result from the complete reflection of light from the hairs, while in species belonging to different families such as *Lycosa* (Lycosidae) and *Josa* (Anyphaenidae), modified cuticular areas acting as reflective surfaces have been identified. Structural colors can function independently or together with pigments in the hairs themselves or in the hypodermis [8]. So far, only three main classes of pigments have been definitively identified in spiders, such as ommochromes (yellows, reds, browns, and blacks) [9], bilins (blue or green pigments) [10], and guanine (contributing to the overall color pattern by creating a white background) [11], which contribute to a wide range of colors and patterns both interspecifically and intraspecifically, as seen in many other arthropods. Hypodermal green pigments have also been found in families with few species such as Araneidae, Dictynidae, and Tetragnathidae [10]. Recent studies have demonstrated that the silver, white, yellow, orange, or red bodies of spiders attract mates, lure prey, or startle predators [12,13,14]. In some crab spiders that ambush on flowers and produce silk ranging from white to yellow depending on the flower’s color, this provides them with a camouflage advantage in capturing their prey [15]. In insects, colors assist in body protection, signaling, and physiological adaptations [16]. Most insects exhibit their colors through the absorption or reflection of sunlight by pigments, cuticular surface structures, or their combination [17]. Many pigment classes are associated with insect coloration. Generally, melanins produce shades ranging from black to reddish brown, while pterins, ommochromes, and carotenoids contribute to red, orange, and yellow colors [18]. Interestingly, some insect taxa have specific pigments; for example, aphids cause the production of various tones in aphids [19], and papiliochromes lead to yellow, orange, and red colors in butterflies [20]. Despite all this diversity, melanins and pterins are two common classes of insect pigments [18]. Pigments are located in specific areas of insect anatomy, such as venation structures in butterfly wings [21] or venation structures in beetle elytra [22]. Therefore, insect colors depend on both pigments and structural features [23]. Additionally, pigments can be produced internally or accumulated from nutrients, as in the case of carotenoids [24,25]. Some special color tones in insects, such as transparent white, golden bronze, iridescent blue, or purple, are solely based on a structural basis [13]. Coloration can also be dynamic, playing significant roles in survival and reproduction [26]. This diversity of colors in insects is important for providing camouflage in some species like ants. Cephalopods (squids, cuttlefish, octopuses), which are among the animals exhibiting coloration in nature, are controlled by three different types of pigment cells, named melanophores, iridophores, and leucophores, and skin pigmentation serves as an effective adaptation mechanism used for various purposes, such as hunting, escaping, communication, and camouflage [4]

Nature is filled with many organic and inorganic substances that can be extracted and transferred to different surfaces. These materials include ochre, which consists of clay minerals and iron oxides, typically ranging in color from light yellow to intense oranges and deep reds [27]; terre verte, a green pigment made from iron silicate minerals [28]; white chalk (white lime), which is obtained by grinding limestone rocks and provides a white color [29]; and lapis lazuli, a valuable stone with a deep-blue color [30]. Therefore, it is important to distinguish coloring agents from colored materials. Coloring agents are substances that extract colors from one place and transfer them to another, and they have been used in various works of art, from early paintings to decorations and paintings.

Colored minerals, soils, and ochres have been consistently used throughout human history. These natural substances are suitable for a wide range of applications, from body adornment to painting, and have been used by all civilizations. One of these is the Cochineal beetle (*Dactylopius coccus* Costa, 1835 (Hemiptera: Dactylopiidae)), especially known for producing a potent red pigment called “carmine”, providing one of the most vibrant red colors obtained from a natural source. This pigment is considered a valuable material for the industry. This material has been used by the Aztec and Maya peoples of North and Central America for a long time. After Hernán Cortés conquered the Aztec Empire in 1521, it was exported from Mexico to Europe, India, and the Far East by Spain. The Cochineal beetle has become an indispensable part of the art world. The dye obtained from this insect has been used to create textiles, paintings, and textile dyes. However, the Cochineal beetle and these special pigments have not only supported artistic expression but have also enchanted and inspired artists [31]. During the Renaissance period, artists preferred the carmine pigment produced by the Cochineal beetle for its bright and vivid red color. Famous painters such as Anthony van Dyck, Raffaello Sanzio di Urbino, and Joseph Mallord William Turner created impressive works by using carmine pigment in their portraits and other paintings.

This red pigment, called carmine, is formulated by crushing cochineal insects. The recovery and purification of carminic acid from raw cochineal is regarded as a difficult and complicated process. Conventional industrial cochineal processing is a multi-step procedure, consisting of treatment with organic solvents, alkaline extraction, solid–liquid separation (flocculation and filtration), insoluble lake formation, recovery of the precipitate (centrifugation), resolubilization of carminic acid, and concentration [32]. However, with the Industrial Revolution, such natural red pigments became obsolete and were replaced by synthetic chemical compounds [32].

This study explores the history of the Cochineal beetle, its role in art, and how it is perceived in the modern world. It delves into the Cochineal beetle, the pigments derived from it, and the famous paintings created using these pigments. This study also examines how Cochineal beetle pigments have found a place in the art world and how artists have utilized this extraordinary source to create unique works.

## 2. The Importance of Insects and Cochineal Beetle *Dactylopius coccus* Costa, 1835 (Hemiptera: Dactylopiidae)

Insects are the most populous within the phylum Arthropoda. With a species count exceeding one million, they stand as the most diverse group of organisms on Earth. Found nearly everywhere on the planet, insects can sometimes be observed in highly dense populations [33].

Insects are indispensable to human life due to their involvement in the pollination of plants, utilization of their products (such as obtaining honey and silk, and in some societies, the widespread consumption of certain insect species as food), and their participation in natural and biological control. Moreover, insects serve as a significant food source for many animal species due to their high protein content and nutritional value. Particularly, birds, reptiles, amphibians, fish, and other vertebrates feed on insects. They also play a crucial role in maintaining balance within ecosystems. Additionally, insects contribute to the aesthetic aspects of art, being used in various forms that are visually appealing. For instance, the wings of butterflies contain microscopic scales that reflect light to create their colorful patterns. Their symmetry, artistic designs, and graceful fluttering abilities make butterflies highly visually appealing insects [34]. Insect parts have been used creatively in Native American crafts, resembling feathers. The Jivaro people of Ecuador have incorporated insect parts into earring designs. Ancient Egyptians chose the scarab beetle as a symbol of sun deities, while ancient Greek coins featured depictions of bees. In various branches of art worldwide, insects have been showcased, including on postage stamps. All these factors greatly amplify their importance. The intricate roles that insects play in ecological systems, coupled with their impact on human societies and the artistic expressions they inspire, underscore the unparalleled significance of this diverse and abundant class of organisms [35].

Furthermore, lacquer (lac) floor finishes, shoe polishes, insulators, various seals, printing inks, and varnishes are significant components in many products obtained from the *Kerria lacca* (Hemiptera: Coccoidea: Kerridae) insect species, which inhabits the tropical regions of South and Southeast Asia. Moreover, several scale insect species provide pigments used for coloring cosmetics, medicines, and beverages. About 40% of beeswax, derived from bees, is utilized in the production of cosmetics, such as lotions, creams, and lipsticks [35].

Cochineal is a vibrant red pigment derived from the body of the female scale insect *D. coccus*, which resides on a cactus plant [35]. Cochineal is native to Central America, with some populations originating from South America, while others come from North America, including Mexico and the southwestern United States. Molecular phylogenetic studies based on gender indicate that *D. coccus* originated in South America and was introduced to Mexico through agricultural products in the pre-Columbian period [36]. It is noted that Cochineal has been used in Mexico since around 1000 B.C., recognizing the significance of this insect as a crucial source of red color. In various languages, the red-colored natural dye derived from the Cochineal is referred to as “kermes” in English, “Cochenille” in German, “Cochenille domestique” in French, “kokkinili” in Greek, and “Cok-neaL” in Arabic [37].

## 3. Definition

The Cochineal insect (*D. coccus*) is a member of the Dactylopiidae family [24]. Adult females are approximately 6 mm in length, 4.5 mm in width, and 4 mm in height. Each insect weighs around 45 mg, thanks to the protective white wax layer that shields them from moisture loss and rain. Similar to other cochineals, young males have wings, while adult females do not [36,38,39].

In contemporary times, the Carmine red insect, *D. coccus*, thrives as a cosmopolitan species in tropical regions where *Opuntia cacti* (prickly pear) grow. These areas typically experience temperatures ranging from 24 to 28 °C, have relatively low humidity, minimal rainfall, and sudden temperature changes. In Europe, such conditions are only found in the Canary Islands [40]. *D. coccus* has adapted to the climate in various regions of the Mediterranean Basin, including Algeria, France, Morocco, Spain, and some parts of Turkey. While attempts to cultivate it have been made in Sicily and Sardinia, success has been elusive. In the late 20th century, a project supported by the European Community in Sicily achieved success in cultivating Cochineal in a protected environment for natural dye production [40]. Presently, these insects are distributed in many parts of the world. Their habitats include dry environments, such as forests, grasslands, cultivated areas, disturbed lands, cactus plants, wild grasses, and gardens [36].

**Figure 1:**
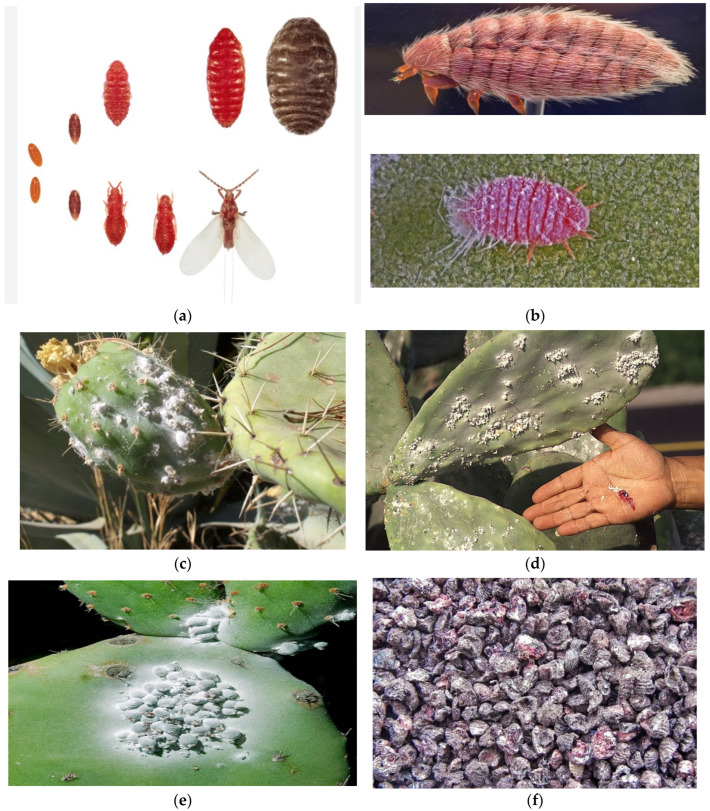
Family: Dactylopidae [43] (**a**) The carmine cochineal (*Dactylopius coccus* Costa, 1835) is an insect belonging to the Dactylopiidae family, and the first age young forms have a red body due to the presence in their blood, called hemolymph, of carminic acid © Jose Mª Perez Basanta [36] (**b**); Cochineal insects’ population beneath the excretion seeping on cacti [44] (**c**); [45] (**d**); the Carmine cochineal (*Dactylops coccus*) lives on the Cactaceae belonging to genera *Opuntia* and *Nopalea* [40] (**e**); female *Cochineal* insects [44] (**f**).

The biological periods of the Cochineal insect are influenced by various factors, such as natural enemies, diseases, climatic conditions, and the physiological state of host plants. Climate conditions play a significant role, particularly during the maturation of insects, where temperatures below 20 °C and above 30 °C, as well as rain and wind, can jeopardize their development. Additionally, the age, species, and health of the host plant can impact the rate of development. According to the literature, the developmental period of the insect ranges from 64 to 120 days, depending on these factors (Figure 1). Consequently, the insect can produce 3–6 generations each year [41,42]. These insects inhabit cactus plants (Cactaceae), especially the prickly pear (*Opuntia ficus*-indica), *Cylindropuntia*, and *Grusonia* species. They often gather in large groups in shaded areas and reside on the cushions of cactus plants, where they feed on the plant’s sap [36].

Cochineal is primarily a non-migratory insect (Figure 1a,b), residing in plant species belonging to the *Opuntia* (*Cactus*) genus. This insect produces carminic acid, which serves as a defense against predators [46] and can be extracted from the body and eggs to produce a valuable natural dye known as carmine (also called crimson or cochineal) [36]. Cochineal insects appear as a white waxy layer on the leaves of cactus plants (Figure 1c–e) and, over time, cause the death of plants due to their absorption of the plant’s sap. However, when dried and ground, cochineal insects produce a remarkable red pigment (Figure 1f) [47]. The outer part of *D. coccus* is a waxy gray color, but adult females turn bright red when crushed due to the presence of carminic acid [47]. Carmine color is stable to a wide range of pHs (3.5–8) and has excellent heat and light stability [48]. This active substance is used as a food dye (E120) and in the cosmetic industry. Therefore, it holds economic and historical significance as a primary source of red dye. According to findings, this dye has been used for this purpose in the Americas since the 10th century, leading to the cultivation of this insect species for its product [36]. The organic red pigment discussed in this study is obtained from the Cochineal insect and is still commercially traded, without losing its importance in the present day.

These insects held significant economic value for pre-Columbian societies in the Andes region. The rulers of the Inca Empire stored these insects as materials in their storehouses because they were the sole source of red dye for clothing and other fabrics. Red was considered the color of royalty among the Incas. After the onset of Spanish colonization in the Americas, these insects were globally shipped as a commercial product. The dried bodies of the females of this insect contain approximately 12–16% carminic acid. The obtained carmine is a dark-red color that can be transformed into different shades by adding metal ions or altering the pH; tin salts or acids produce a bright-red color, while iron or alkaline compounds turn it into a dark purple. The Salasacas people in Ecuador still use this process to color their fabrics, especially the red woolen garments worn around the shoulders by women, personalizing them by choosing different tones of red. They likely collected scale insects, probably of the species *D. confusus*, from natural areas, pressed them into pieces, and used them to dye garments in sets of three. One was left red, the second was dipped in lemon juice to turn it red, and the third was rubbed with wood ash to turn it purple [36].

All species of *Dactylopius* owe their color to the presence of carminic acid in their bodies. The amount that can be obtained from dried females of *D. coccus* is higher compared to other species. This type of “biological” coloring is used as an indicator and reagent in histology and bacteriology. Before the emergence of aniline-based, cheaper synthetic dyes, the aqueous solution of the extract from the dried bodies of Cochineal was widely used not only in dyes but also in the pharmaceutical, cosmetic, and food industries. The shades of color, ranging from fuchsia to red and violet, are achieved by adjusting the pH with dyes and mordant modifiers [40].

**Figure 2:**
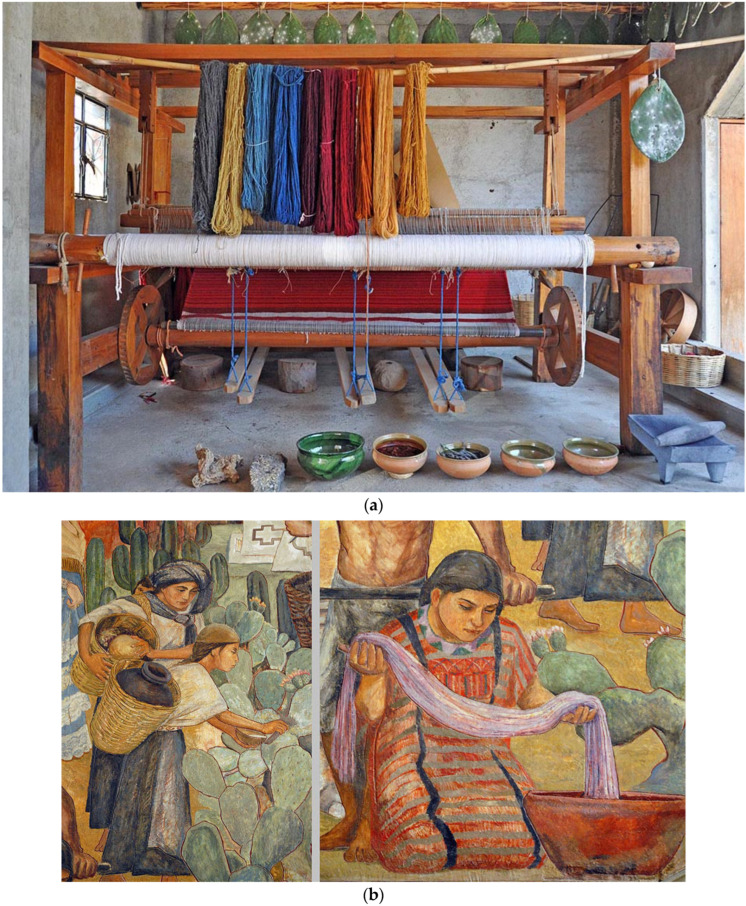
Laboratory with hanging high cladodes, loom and the material for hand crafting with wools of various shades of color obtained varying the bath pH [40] (**a**); interesting mural by Arturo Bastia Bustos in Oaxaca Palacio Museum. It shows the various phases of the productive process from the harvest in the field of the cochineal by the women, up to the coloring of the fabrics with *Dactylopius coccus* in Mexico in pre-Columbian eras © Karen Elwell [40] (**b**).

The Aztecs developed suitable production systems for Cochineal insects to obtain a large amount of raw material. These systems are still partially adopted today in South Africa, India, and Central-South America. Growers collect pre-ovigerous females from various plants before the rainy season and use them to infect the cladodes of plants more suitable for the dry season, thereby promoting the development of Cochineal and restarting production. A more modern production system involves sampling the cladodes of *Opuntia-ficus indica* in the field and transferring them to a protected environment. In this environment, with the help of suitable hooks suspended from supportive structures, Cochineal infection is achieved by placing vegetable fibers containing ovigerous females into a basket in contact with each cladode [40]. The newly produced young forms reach maturity and transform into adult females anchored to the cladode, turning into dried bodies of a dark-gray color in about three or four months. Subsequently, the dusty wax is cleaned, and the dried bodies are pulverized into powder. To obtain one kilogram of dye, it is necessary to pulverize 80,000 to 100,000 adult females that have not yet laid eggs. The powder obtained in this way undergoes a process with water to extract the carminic acid it contains. The resulting dye is then processed with aluminum salts to make the lacquer shinier. Ethanol is added to precipitate the compound and obtain a water-soluble powder. In the past, the main use of the obtained colors was in dye houses where different tones of fuchsia-, red-, and purple-colored fabrics and wool were achieved by regulating the pH of dyes and fixatives (Figure 2). Currently, this color is used to tint cosmetics such as lipsticks and blushes and is also used in the food industry with the code E120 to color yogurts and red fruit juices [40,49].

The cochineal insect has played a significant role in the production and trade of color throughout history. In South America, when discovered by Europeans, this insect, known and used by indigenous peoples for thousands of years, gained great value and had a significant impact on global trade [47]. Historically, while various sources, such as rust, herbs, roots, oil, and even cow dung, have been used for hundreds of years as a pigment source for red dye, none of them could provide the richness and vibrancy that could truly capture the essence of the color. Often, they faded over time, turning into a dull, unappealing brown. However, when the Spaniards reached Central America in the 16th century, this changed. They discovered the red cochineal insect (*D. coccus*), cultivated for centuries by farmers in the southern mountainous regions of Mexico, feeding on cacti. Realizing the opportunity in their hands, the Spanish guarded the knowledge of red cochineal and its sources as a secret. To protect their monopoly, they went to great lengths to collect the red cochineal themselves and began exporting these pellet-like objects to European merchants who had no idea what they were [50]. The Spanish then brought them to Europe [51]. While cochineal and *Opuntia cacti* were exported to Southern Europe and many countries in Africa and Asia, most of the cochineal dye is still produced in Latin America today. Peru is the primary global producer, covering around 85% to 90% of the worldwide market, with much of its production centered around collecting insects from natural Opuntia shrubs in the Andes, especially in the Ayacucho region [52]. Additionally, particularly in Armenia, local dyes produced from the Armenian cochineal insect (*Porphyrophora hamelii* Brandt (Hemiptera: Margarodidae)), which belongs to a different taxonomic family than the cochineal from the Americas, have been abundantly produced since the 8th century BCE and occasionally exported to cities such as Venice, Genoa, and Marseille [47]. Both insects produce red dyes and are commonly referred to as cochineal [53]. However, the cochineal species *D. coccus* from the Americas, which can be harvested several times a year and yields a much more concentrated dye, eventually supplanted the Old World *Porphyrophora* dyes over time [54].

Later on, Cochineal gained significant popularity in Europe in the 16th century, becoming widely used in the textile, dyeing, cosmetic, and food industries. The story of producing pigment from the cochineal insect is often a traditional narrative passed down as a legend or myth. This narrative is set in a very ancient period of human history, illustrating the interactions of gods and how nature took shape. According to the myth, during the time when gods invaded the world, two powerful deities engaged in a deadly battle for a precious species of cactus, and their blood splattered onto the cactus fields during this mortal combat. The siblings, in their attempt to carry their bodies to the sky, descended, but their spilled blood transformed into the tiny Cochineal insects, which became the most sought-after natural dye producers in human history [55]. The historical narrative of the Cochineal insect, rooted in myths, disrupted the red pigment market with the discovery of the West Indies (the Americas). Prior to this, a red dye plant was exported from the New World to Europe, marking the most valuable import after gold and silver. Initially, the red pigment obtained from the kermes insect was in use, but due to the scarcity of kermes insects and the weak intensity of the red color in their pigment, the Cochineal insect became more popular.

The Aztecs enhanced the potency of the natural dye produced by the Cochineal insect by selectively breeding the insect through careful cultivation methods. They prepared agricultural fields with fertilizers and created conditions necessary for the healthy growth of the cactus. The fields were protected from wind, rain, and frost to ensure the stability of the insect’s habitat. Additionally, predatory animals such as spiders, mice, and lizards were kept away to prevent damage from other insects. These meticulous farming and production processes contributed to the increased quality of the Cochineal insect’s natural dye. In modern Cochineal farming, a similar approach has been maintained, following the practices initiated by the Aztecs. Perhaps the only difference is that the process is now conducted in a more controlled manner. The entire life cycle of the Cochineal insect takes three months. Nowadays, commercially cultivated Cochineal insects and cacti are raised in sheds to control water access and regulate temperatures. Since the insects are highly sensitive to both cold and excessive heat, they are maintained at a constant temperature of 27 °C and harvested when they reach adulthood. In traditional harvesting methods, they are manually collected from the cacti using a spoon, but in modern methods, pressurized air is employed to remove the insects. The process of making cacti spineless is also carried out to facilitate harvesting. In nature, the insect is naturally propagated from plant to plant by the wind. In modern Cochineal farming, controlled reinfestation is employed to multiply the insects. Commercial producers use various methods for this process. Some farmers manually transfer the red insects onto healthy plants using fox-hair brushes, while others create pockets called Zapotec nests on the cacti to provide a habitat for the insects, allowing them to adhere more easily. The global significance of the Cochineal insect persisted for 300 years until the mid-19th century. The cultivation of Cochineal insects began to decline as the availability of cheaper products increased with the introduction of synthetic dyes.

## 4. The Use of Red Color in the Art of Painting

**Figure 3:**
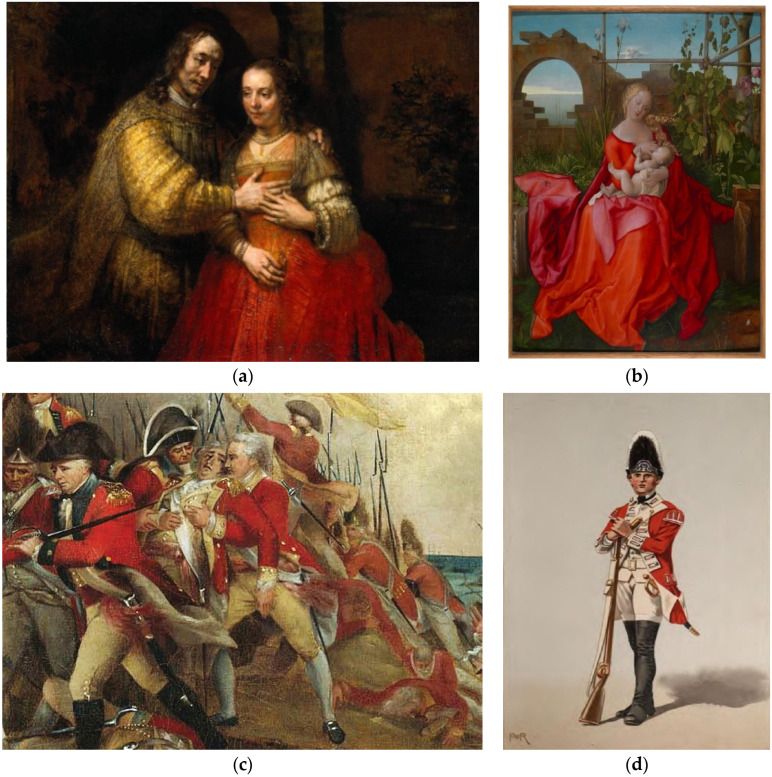
About 1665–1669, Rembrandt Harmensz. van Rijn. Oil on canvas, 121.5 cm × w 166.5 cm. Rijksmuseum, Amsterdam. On loan from the City of Amsterdam (A. van der Hoop Bequest) [56] (**a**). The vibrant red obtained from Cochineal in Europe was like nothing else. An example is Albrecht Dürer‘s workshop, particularly the Lily Madonna, dated around 1500–1510 [57] (**b**); English military uniforms, including some iconic red jackets, were dyed with cochineal, providing an advantage in concealing bloodstains to some extent [57] (**c**); only the jackets of officers were dyed with cochineal, giving them a scarlet color [57] (**d**).

From Ancient Egypt to the present day, the acquisition of the color red in the art world has involved various methods. Ancient Egypt stands out as a pioneering civilization in the use of red pigments. Oxides and natural minerals were employed to obtain red-colored paints during this era. During the Roman Empire period, red pigments were extensively used, particularly in the red paints used in frescoes and mosaics, obtained from red oxides and stones. In medieval Europe and the Renaissance, the color red held particular importance, especially in works created for the church. During this period, the red pigments used contained toxic substances such as red mercury and sulfur. The Renaissance was a time when artists developed new techniques to achieve more complex shades of red. Artists in the 16th century discovered that Cochineal, like other organic dyes obtained from plants and insects, could be transformed into a useful pigment. The pigment made from Cochineal was called “carmine”, and renowned artists such as Raphael and Leonardo da Vinci were among those who used this pigment. “Artists often created a pigment called ‘lake’ by mixing Cochineal with a binder. Naked eye observation makes it impossible to determine which painters used Cochineal to create their reds. However, recent advancements in chemical analysis have confirmed the use of Cochineal in many famous works. One such work is Rembrandt van Rijn‘s painting ‘Jewish Bride’” [56] (Figure 3).

Just as Anthony van Dyck depicted shining red silk fabrics in works like “Charity” or “Portrait of Agostino Pallavicini,” other painters of the time also used Cochineal red to portray red fabrics. These bright reds were utilized to create the richness and splendor of the clothing worn by the aristocrats of that era. Therefore, Cochineal insects had a significant influence on the artworks and fashion of this period [56].

Turner is another painter who utilized the striking color of Cochineal insects. However, there is a significant drawback to using this pigment. Unlike Cochineal dye, which generally retains its color, Cochineal pigments in paintings tend to fade when exposed to light. This effect is particularly pronounced in watercolors. In the watercolor works of J.M.W. Turner, this phenomenon is clearly visible. Landscape paintings that initially started with intense Cochineal red have faded over time due to the effects of exposure to light. This fading has posed a challenge to artists who desire to capture the enduring vibrancy of Cochineal red in their paintings.

**Figure 4:**
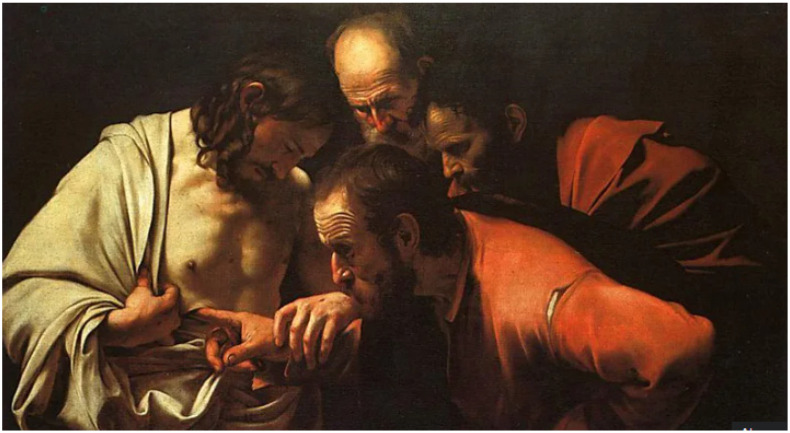
Caravaggio, “The Incredulity of Saint Thomas”, 1602, Oil on canvas, 107 cm × 146 cm (“The Incredulity of Saint Thomas”) [59].

Similarly, López de Arteaga‘s undated work “The Incredulity of Saint Thomas” (Figure 4) pales in comparison to Caravaggio‘s rendition of the same subject. However, the red robe worn by Jesus in López de Arteaga‘s painting, signifying his holiness, certainly stands out on the canvas. Both artists employed cochineal, which helped to establish the characteristic dramatic contrast of the Baroque style [58].

## 5. Conclusions

The use of insect-derived paints in the art of painting is considered an intriguing material and technique, especially among old masters. Paints obtained from insects are generally regarded as a natural and organic source, making them an environmentally friendly option. Additionally, the colors and textures of these paints often differ from commercial ones, adding uniqueness to artworks. Incorporating insect-derived paints into artworks allows for the integration of an organic element sourced from nature. Furthermore, the use of these paints can help enrich artworks aesthetically and conceptually. In conclusion, the use of insect-derived paints depends on the artist’s preferences and the messages they aim to convey through their works.

## Data Availability

No new data were created or analyzed in this study.
